# Tumor Microenvironment, a Paradigm in Hepatocellular Carcinoma Progression and Therapy

**DOI:** 10.3390/ijms18020405

**Published:** 2017-02-14

**Authors:** Maryam Tahmasebi Birgani, Vinicio Carloni

**Affiliations:** 1 Department of Medical Genetics, School of Medicine, Ahvaz Jundishapur University of Medical Sciences, Ahvaz 63461, Iran; maryam_tahmaseby@yahoo.com; 2 Department of Experimental and Clinical Medicine, University of Florence, Largo Brambilla 3, Florence 50134, Italy

**Keywords:** cancer therapy, hepatocellular carcinoma, tumor microenvironment, immune cells, fibroblast cells, endothelial cells, extracellular matrix

## Abstract

Hepatocellular carcinoma (HCC) is among the most lethal and prevalent cancers in the human population. Different etiological factors such as hepatitis B and C virus, alcohol and diabetes cause liver injury followed by inflammation, necrosis and hepatocytes proliferation. Continuous cycles of this destructive–regenerative process culminates in liver cirrhosis which is characterized by regenerating nodules that progress to dysplastic nodules and ultimately HCC. Despite its significance, there is only an elemental understanding of the pathogenetic mechanisms, and there are only limited therapeutic options. Therefore, the study of the involved molecular mechanisms can open a new insight to define more effective treatment strategies. A variety of alterations have been reported in HCC patients, particularly the cancer-associated microenvironment components including immune cells, fibroblast cells, endothelial cells and extracellular matrix can support the neoplastic cells to proliferate, growth and invade. This review summarizes the current state of knowledge and highlights the principal challenges that are relevant to controlling this milieu.

## 1. From Normal Liver to a Malignant Liver

**Figure 1:**
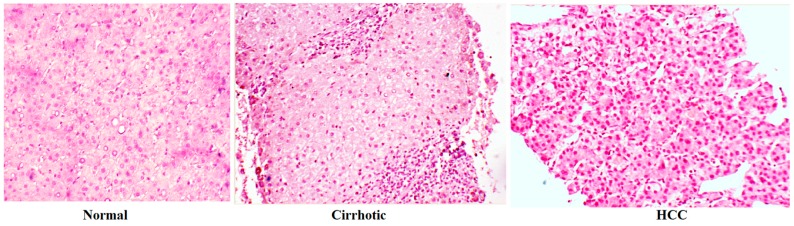
Histological aspects of normal liver, cirrhotic liver and hepatocellular carcinoma (HCC). Magnification, 100×.

Structurally, the liver consists of five parts: (1) hepatocytes and hepatic lobule; (2) vascular system; (3) hepatic sinsusoidal cells; (4) biliary system; and (5) stroma [1]. Hepatocytes are parenchymal cells that metabolize or detoxify all the substances which are absorbed by portal vein from the gut. Hepatocytes occupy around 60% of totally cells in the liver [2,3]. On the other hand, non-parenchymal parts including endothelial cells, Kupffer cells, stellate cells and lymphocytes are mostly attributed to the immune modulatory function of liver in the body in front of invading pathogens [3]. Once hepatic injury incurred, the liver histologically changes to a fibrotic tissue. Fibrosis is characterized by abnormal liver nodule formation surrounded by collagen fibrils which are secreted from activated hepatic stellate cells, during fibrosis the liver parenchyma is irreversibly replaced with collagen-rich scar tissue [4]. Notably, in acute injuries, liver reverse the injury due to its capacity to repair the damaged tissue while in chronic condition the healing processes fail [5]. Cholangiocarcinoma (from the epithelium of intrahepatic bile duct), hepatoblastoma (from hepatic precursor cells) and hepatocellular adenoma (from hepatocytes) are rare forms of liver cancer [6]. Around 85% of primary liver cancers diagnosed as HCC are developed as a result of chronic hepatitis caused by HBV (hepatitis B virus) HCV (hepatitis C virus), or NASH (non-alcoholic steatohepatitis) [3,7,8,9,10]. Therefore, studies of the underlying mechanisms of hepatocarcinogenesis are mandatory to find an effective HCC therapy (Figure 1) [11,12,13,14,15,16,17,18].

## 2. The Most Reported Signaling Pathways in Hepatocellular Carcinoma

**Figure 2:**
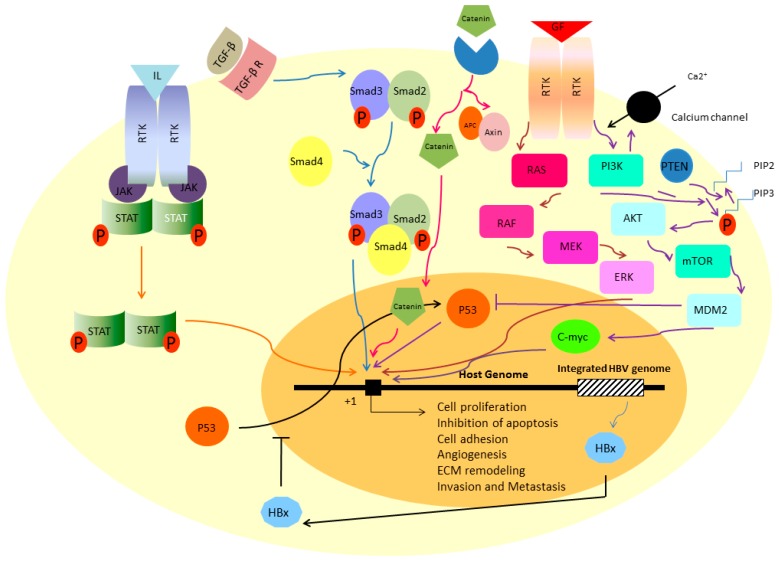
The most reported signaling pathways in HCC.

Whittaker has recently discussed the well-identified signaling pathways during the HCC progression [19]. These include RAS/RAF/MEK/ERK, PI3K/AKT/mTOR, HGF/MET, FGF, IGF, JAK/STAT, p53 and TGF-β signaling pathways [19]. In the first part of this review, we briefly discuss the importance of some of these paths in HCC to make sense how a normal liver can swap towards the malignant one (Figure 2).

## 3. Ras/Raf/MEK/ERK Signaling Pathway

The path is one of the key signaling cascades in the cells where the cooperation of the proteins Ras, Raf, MEK and ERK eventually regulate the proliferation, differentiation and apoptosis. Extracellular signals are usually growth factors (including EGF, FGF and PDGF), hormones or differentiation inducers [20]. The cascade begins with binding of a ligand to the surface receptor tyrosine kinase (RTK). Following the attachment, autophosphorylation can occur on tyrosine residue of RTK cytoplasmic tails which then activate the Ras, Raf, MEK and ERK sequentially. ERK goes to the nucleus and triggers the expression of genes of proliferation (Figure 2). Constitutive activation of the cascade due to the mutations, is critical factor to stimulate the hepatic stellate cells (HSC) towards fibrogenesis and myofibroblast phenotype which will be explained later [5]. There are several lines of evidence showing the importance of Ras/Raf/MEK/ERK signaling pathways in liver cancer progression, especially HCC [20]. Overexpression of c-Raf, MEK, and ERK was observed in more than half of HCC patients [21]. Schmitz and coworkers also showed the activation of ERK-1/2 in HCV positive-HCC samples which was significantly associated with aggressive phenotype of the cells [22].

## 4. PI3K-AKT-mTOR Signaling Pathway

Phosphatidylinositide 3-kinases (PI3K) is another target of RTKs (after RTK-ligand interaction) which phosphorylates the phosphatidylinositol (4,5)-bisphosphate (PIP2) to phosphatidylinositol (3,4,5)-trisphosphate (PIP3). The pathway is reversed by tumor suppressor PTEN. In unstimulated normal cells, PIP3 level is very low [23]. The mTOR (Mammalian Target of Rapamycin) is functionally involved in cell growth and metabolism [24]. In fact, the mTOR is energy sensor and activates the cellular translation and lipogenesis during the nutrition rich condition while it is inhibited due to hypoxia or DNA damage (Figure 2) [25]. Deregulated PIP3-AKT-mTOR was frequently observed in HCC patients [26]. In a study by Chen, overexpression of AKT and mTOR was found to be correlated with HCC invasion and metastasis [27]. It has been demonstrated that AKT activation is correlated with reduced overall survival in HCV positive patients suffering from HCC [22].

## 5. TGF-β Signaling Pathway

Transforming growth factor-β (TGF-β) is one master regulatory system in the cells to modulate the process of proliferation, death, cytoskeleton orchestration, cellular adhesion and wound healing in a cell specific manner [28]. The superfamily of TGF-β consists of more than 30 proteins including TGF-β isotypes, myostatin, activin, inhibin, nodal and bone morphogenetic proteins. All of these ligands are synthesized as latent precursors and will be activated through a proteolytic reaction. This is one of the several ways in which the path can be regulated [29]. As the ligand is activated, it can bind to the TGF-β receptors and transfers the signals into the nucleus through co-Smad complex (Smad2, Smad3 and Smad4 proteins) [30]. Although the role of TGF-β in human cancer is controversial, its involvement in fibrotic responses has been well documented. In this way, matrix deposition or recruitment of immune cells at the site of inflammation are two of several important actions of TGF-β cascade [30]. Paik and coworkers confirmed that the expression levels of TGF-β receptors have been downregulated in HCC samples in comparison to the adjacent normal tissues showing its importance for HCC initiation. This downregulation was also associated with higher tumor size and proliferation capacity [31]. The gene expression alterations were also observed in co-Smad subunits. As an example, increased expression of Smad4 was observed in HCC tissues and its siRNA-mediated suppression inhibited the colony formation in Huh7 and PLC cell lines [32].

## 6. JAK/STAT Signaling Pathway

To transfer the signal of growth factors and inflammatory intermediates including interleukins and interferons, JAK/STAT signaling has been well established which regulates proliferation, differentiation, apoptosis and in one word, tissue homeostasis [33]. Once the ligand attached, dimerization of receptors occurs which recruits the STAT proteins near the membrane through the JAK proteins. Now, STATs can be phosphorylated by JAK and moved to nucleus where they can act as transcription factor of gene expression (Figure 2) [33]. It has also been demonstrated that following hepatectomy, JAK/STAT will be activated through TNF-induced SOCS-3 overexpression [34]. Similar to a variety of human malignancies, HCC tissues also show the disturbed JAK/STAT signaling pathway. The path is one of the key activators of HSCs cells whose role in HCC progression has been demonstated repeatedly. Inhibition of JAK/STAT signaling decreases the proliferation, migration and ECM-producing characters of HSCs while triggers their apoptosis [35]. Saxena et al. found that leptin increased the invasiveness and migratory potential of HepG2 line via JAK/STAT activation [36]. In a microarray analysis, Basu et al. showed that HCV core-transfected hepatocytes can also induce the STAT signaling through overexpression of IL-6, STAT3 and leptin receptor [37].

## 7. β-Catenin Signaling Pathway

β-Catenin signaling pathway is one of the most active signaling pathways in hepatocytes playing an important role in liver development and of course regeneration [6]. Similar to other signaling pathways, the pathway starts with binding a β-catenin to its cognate receptor and transfers a signal into the cells where it can act as transcription factor of TCF-response promoters. When the receptor is free of cargo, β-catenin is phosphorylated by complex of APC, Axin and GSK3 and is degraded following ubiquitination [6].The importance of Wnt pathway in progression of liver cancer has been demonstrated in several studies. Constitutive expression of β-catenin and Axin has been observed in HCC patients [38]. Following HBx-transfection, β-catenin has been stabilized and translocated into the Huh7 nucleus [39].

## 8. Signaling Pathway of p53

The tumor suppressor p53 is frequently mutated in half of human tumors. It is induced through the cellular damages including hypoxia or viral infection and eventually leads to promote the cell cycle arrest or apoptosis [40]. The p53 loss of function following mutations has been frequently observed in precancerous dysplastic nodules and plays an important role from passing this stage toward early HCC [19]. It has also demonstrated that codon 249 of p53 is preferentially mutated due to exposure to aflatoxin B1 or hepatitis B or C infection. This mutation is G:C to T:A and has been detected in serum of HCC patients [41]. Honda et al. showed that altered expression of p53 has been correlated with pathological features of the disease such as histological grade, survival, response to the therapy [42].

## 9. Viral Infections Disrupt Normal Signaling Pathways

It is well-documented that viral replication can interrupt the genomic stability as integrated into the genome. For example, as hepatocytes are infected with HBV, the virus-coding protein HBx avoids the p53 to interact with repair system-associated proteins by blocking its entrance into the nucleus so accumulates the DNA errors [43]. HBx protein also interacts with other transcription factors including TFIIB and TFIIH, CBP, PKC, NFκB, Ras, Raf, MAPK, AP-1 and JAK/STAT which are normally involved in processes of cell growth or apoptosis [43]. Interestingly, it has been recently found by Liu that hTERT expression and telomerase activity are increased in HBx-transfected cells and HBx-positive HCC samples [44].

## 10. Tumor Microenvironment: New Horizon in Hepatocellular Carcinoma Pathogenesis

**Figure 3:**
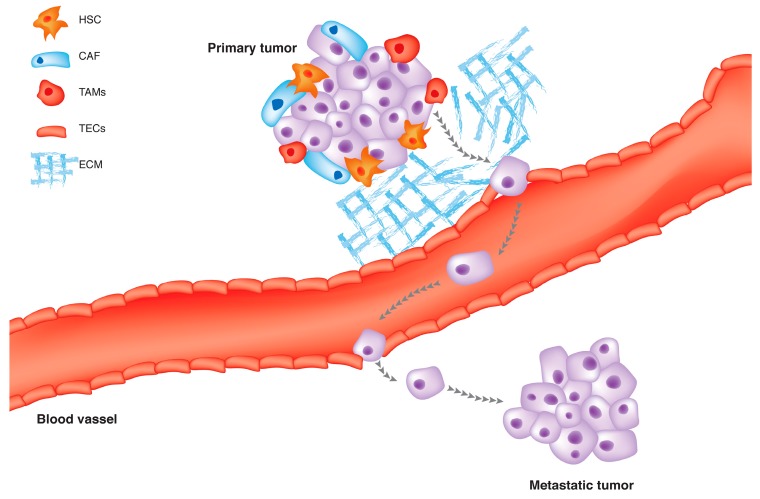
The tumor microenvironment (TME) is the cellular milieu in which the HCC tumor grows, including surrounding blood vessels, hepatic stellate cells, macrophages, lymphocytes, cytokines, chemokines and the extracellular matrix (ECM).

Normally, stroma maintains the tissue homeostasis and acts as a barrier toward tumor formation; however, when a cell starts to be cancerous, its surrounding matrix changes in a way to support cancer development [45,46]. This modified stroma around the malignant cells is termed tumor microenvironment (TEM) [47]. The architecture of a typical TEM is composed of fibroblasts, myofibroblasts, endothelial cells, pericytes, adipose cells, immune and inflammatory cells, and the extracellular matrix (ECM) elements (Figure 3) [8,45]. It has also enriched with diffusible cytokines, chemokines or enzymes which are secreted from both cancerous and noncancerous cells. In a such milieu, the reciprocal crosstalk of all these compartments with each other eventually decides how the tumors growth [48]. There is a lot of evidence showing that HCC initiation and progression beneficiate from its associated tumor territory which will be discussed in the following sections. In the first line, we focus on structural and functional elements of TEM to explain how these components can influence HCC progression and metastasis. Besides, how this tumor niche may be exploited to treat HCC will be addressed.

## 11. Carcinoma Associated Fibroblasts (CAFs)

Fibroblasts are elongated cells with spindle-shape morphology which are embedded in fibrillar matrix of the connective tissue. They are actively involved in wound repair, deposition of extracellular matrix (ECM), tissue maturation and inflammatory responses [49]. A growing body of evidence indicates that a sub-population of fibroblasts can modulate cancer progression. These cells are known as carcinoma-associated fibroblasts (CAFs) or tumor-associated fibroblasts (TAFs). The CAFs have been extracted from a variety of human tumors including prostate [50], breast [51], ovary [52] and esophagus [53]. In tumor tissue, CAFs are activated from normal fibroblasts, although they can also be originated from endothelial cells, epithelial cells, smooth muscle cells, pre-adipocytes and bone marrow-derived progenitors [46]. The CAFs are phenotypically and genetically different from the ancestral cells. They express α-smooth muscle actin (α-SMA), a marker of myofibroblasts [54]. Since HCC tumors are initially arisen in the context of cirrhosis where the amount of activated fibroblasts are impressive, it is not so far-fetched that CAFs influence the HCC progression [55]. How the resting fibroblast cells moved to the activated state (myofibroblasts) is not clear but three probable models were suggested by Shimoda et al.: (1) mesenchymal cells trans-differentiate to myofibroblasts; (2) specialized circulating progenitor cells such as fibrotic cells or mesenchymal stem cells comes to the side of tumor and differentiated to myofibroblasts; (3) myofibroblasts can be originated from the rare population of pre-existing myofibroblasts which are clonally expanded during tumorigenesis; and (4) genetic alteration within stromal cells leads to formation of myofibroblasts and a variety of chromosomal aberrations, somatic mutations and epigenetic modifications were also observed in stromal region and micro-dissected from different human tumors although the results are controversial [46]. Phenotypically, CAFs have prominent Rough endoplasmic reticulum(R-ER) and Golgi apparatus makes them suitable for protein synthesis such as ECM constituents. As an example, type I collagen, fibronectin and tenascin-C and SPARC (secreted protein acidic and rich in cysteine) [49]. The CAFs remarkably secrete matrix-metalloproteinases including *MMP-2*, *MMP-3* and *MMP-9*. This feature gives the cells to remodel the ECM and facilitates the tumor invasion through digesting ECM barriers and escape from primary tumor site [54,56]. Additionally, CAFs are potent to modulate the immune response by infiltrating the monocytes and macrophage to the site of injury through the secretion of a variety of cytokines and chemokinessuch as MCP-1 (monocyte chemotactic protein-1) and IL-1 (interleukin-1) [49]. By secreting the SDF-1 (stromal cell-derived factor-1), CAFs recruit the endothelial progenitor cells into the tumor site and promote angiogenesis [51]. It is very important to stress that CAFs remain active even if the stimuli are removed. This is contrary to the process of wound healing, where the activated cells undertake apoptosis or nemesis [49,57,58]. Regarding to acquisition of these features by CAFs, scientists believe that these cells are one of the key modulator of tumor initiation, progression, metastasis and invasion. Tissue culture experiments alongside with in vivo xenograft models play in important role in this way. The researchers found that tumor growth, angiogenesis and metastasis increased when the cancer cells are co-implanted with CAFs and not with normal fibroblasts into the nude mice. As an example, when Ras-transformed MCF-7 breast cancer cells are co-injected into the nude mice with CAFs or normal fibroblasts, xenograft infused with CAFs grows larger than xenograft infused with normal fibroblast cells [51]. A growing body of evidence showed the importance of CAFs during HCC progression. It is demonstrated that the frequency of CAFs around HCC region is positively correlated with the tumor size. Additionally these cells secrete the hepatocyte growth factor (HGF) in a level higher than the normal fibroblasts [59].CAFs-secreted CCL-2, -5, -7 and CXCL16promote the migration and invasion of HCC cells and facilitate their metastasis to the bone, brain and lung in severe combined immunodeficiency SCID mice by activation of TGF-β signaling pathway [60]. Tuanjie and his colleagues found that fibroblast cells suppress the NK cells function by secretion of prostaglandin E2 [61]. It is necessary to mention that natural killer dysfunction was observed in several human solid tumors [62].

## 12. Hepatic Stellate Cells (HSCs)

Hepatic stellate cells (HSCs) or Ito cells are non-proliferating cells are localized in basolateral surface of hepatocytes and the anti-luminal side of sinusoidal endothelial cells where they can easily contact with hepatocytes and endothelial cells [5]. They are morphologically spindle-shaped, with elongated nuclei and retinoid-storing droplets in their cytoplasm [63]. Hepatic stellate cells act as critical effectors during liver injuries [63,64]. As a consequence of liver fibrogenesis, HSCs trans-differentiate into the myofibroblast-like cells and became more contractile, proliferating and potent to synthesize extracellular matrix components [65]. Activation of HSCs occurs in three phases, initiation, perpetuation and resolution. During these steps, HSCs are exposed to the stimuli from sinusoidal endothelium, Kupffer cells, hepatocytes, platelets and all products of injured hepatocytes. Cells then moved into the site of injury and release the pro-inflammatory, pro-mitogenic and pro-fibrogenic factors eventually lead extracellular matrix accumulation and stroma remodeling [5,66]. Infiltration of the activated HSCs into the stroma and their localization around tumor sinusoids, suggests that HSCs may be involved in HCC progression [67]. Tumoral hepatocyte conditioned media significantly increased the proliferation of rat HSCs as well as increased expression of α-SMA, Desmins, PDGFR and Gelatinase A secretion [68]. Amann and colleagues examined the impact of HSCs on HCC progression. They found that collected conditioned media from HSCs increased the growth and invasiveness of HCC cancer cell lines. Similarly, co-implantation of HSCs along with HCC cells into the nude mice increased the tumor growth and invasiveness through activation of NFκB and ERK signaling pathways [69,70]. Additionally, data were obtained from microarray analysis, revealed the activation of several genes of inflammation, chemotaxis, angiogenesis and metalloproteinase following the co-culture of hepatoma cells with activated HSCs [9]. In an orthotopic liver tumor mouse model, Zhao et al. demonstrated that HSCs provide an immunosuppressive niche for HCC through induction of regulatory T cellsand myeloid-derived suppressor cells (MDSCs) probably due to activation ofCOX2-PGE_2_-EP4 signaling pathway [71,72].

## 13. Immune and Inflammatory Cells

The association of inflammation and cancer was firstly hypothesized by Rudolf Virchow observations in 1863 as chronic irritation theory. In this theory, cancer is caused by severe irritation. Virchow found that certain cancers are associated with inflammatory macrophages [73]. In later years, other researcher showed that many cancers (including lung, prostate, gastric, colorectal, bladder, hepatocellular carcinoma, pancreatic, cervical, esophageal, ovarian and melanoma), but not all are connected with inflammation [74,75]. The Virchow’s theory was powered as scientists found that anti-inflammatory drugs, such as aspirin, inhibit tumor formation and development [76,77].

In 1986, Harold Dvorak believed that the tumor environments are very similar to the wounds in some aspect. He said that tumors are actually “wounds that do not heal” [78]. Now, it is uncovered that tumor inflammatory cells come to the tumor area and prepare a niche for the neoplastic cells and facilitate cancer angiogenesis, metastasis and invasion [79]. In fact, secreted cytokines and chemokines by cancer cells recruit the immune and inflammatory cells to the site of neoplasm. Neutrophils, monocytes, lymphocytes, dendritic cells, eosinophils and mast cells are the commonly observed cells in tumor stroma although their count depends on cancer type [73,74]. It is interesting to know that tumor-associated inflammatory cells are different from those classically related to inflammatory pathways with tumor destructive action. Most of these cells are immature which are confined in tumor mass or invasive edge. Similar to cancer cells, the inflammatory cells also secrete lots of cytokines, chemokines, and proteases affecting cancer development [73]. Here we focus on macrophages as they are one the most prominent inflammatory cells in tumor stroma and discuss their role during cancer progression especially in case of HCC.

## 14. Tumor-Associated Macrophages (TAMs)

**Figure 4:**
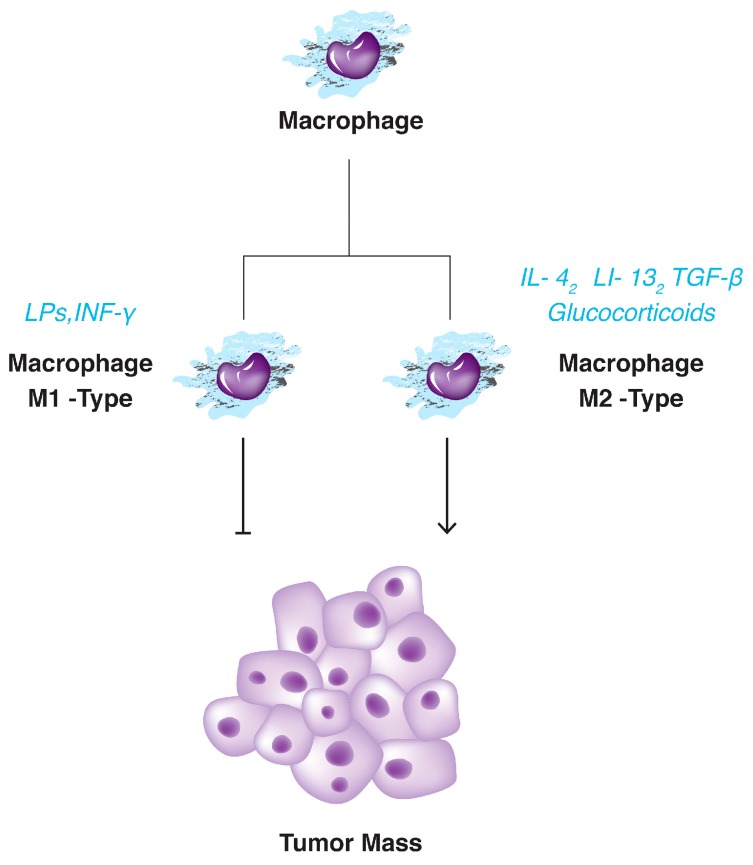
Tumor associated macrophages (TAMs) are different from conventional macrophages which act againts tumor formation.

Macrophages are a type of leukocytes with antigen presentation capacity and are actively involved in tissue remodeling, phagocytosis and scavenging the foreign substances or cellular debris [80]. They are originated from the circulatory bone marrow monocytes or yolk sac and are localized in the tissue [81]. When the macrophages come around the tumoral region, these cells are termed tumor-associated macrophages (TAMs). It is demonstrated that tumor-surrounding TAMs play an important role during tumor development [81]. Increasing number of studies showed that macrophages facilitate cell proliferation, angiogenesis, metastasis and invasion [79]. However, a paradox quickly comes to the mind, how do the antitumor macrophages cause tumor growth? Beside, how do they reach into the tumor boundary? Macrophage balance hypothesis provide an explanation to the questions. Based on the theory, two distinct phenotypically states are related to the macrophages, differing in function and the produced cytokines (Figure 3).These phenotypically changes are called macrophage polarization [77]. The classically activated macrophages or M1-type are induced by Th1 cytokine INF-γ or microbial antigens such as Lipopolysaccharides (LPSs). They are classically macrophages that exert their cytotoxic function by releasing the reactive oxygen species (ROS) or toxic intermediates [62]. The second one is the alternatively activated macrophages or M2 with low antigen-presenting capacity and polarized by Th2 cytokine IL-4 or IL-13, TGF-β or glucocorticoids (Figure 4) [82]. In spite of M1 macrophage, M2 cells decreased the inflammation and promote tissue repair. It seems that in the early stage of tumorigenesis, M1 macrophages eliminate the tumor cells as soldiers of adaptive immunity. However, in advanced stages, M1 macrophages replaced with M2-type. M2 macrophages suppress the adaptive immune system and promote the cancer proliferation, angiogenesis and ECM remodeling as well. Eventually, tumor cells escape from the immune barriers and invade [69,77]. In other word, Th2-induced macrophage polarization changes the anti-tumor environment to the immunosuppressive niche which is adaptive to the tumor cells [81]. It is demonstrated that most of the tumor cells express the protein termed monocyte chemotactic protein-1 (MCP-1) who recruited the TAMs into the tumor stroma although we should not ignore the crucial role of tumoral M-CSF (macrophage-colony stimulating factor), angiopoietin-2, VEGF (vascular endothelial growth factor) and MIP-1α (macrophage inflammatory protein 1α) in this way [74,83]. Usually, TAMs are accumulated in hypoxic region of the tumors where they are more prone to produce the pro-angiogenic factors such as VEGF, TNF-α and matrix metalloproteinases [84]. Pro-angiogenic factors interact with ECM and digest its elements such as fibrin and collagen to remodel ECM in favor of vessels formation and of course cancer extravasation [81,82,85]. Like other cancers, liver malignancies are also benefited from TAMs at the site of injury [82]. Dong et al. showed that high presence of M2 macrophage are associated with aggressive phenotype of HCC [86]. Besides, monocytes count and serum level of IL-6 were significantly higher in HCC patients [87]. On the other hand high level of pro-metastatic cytokines including IL-6, IL-1 and TNF-α were significantly higher in the blood samples of HCC patients than those from healthy counterparts [88]. As previously mentioned M2 macrophages are strongly release all three cytokines. Peng and coworkers showed that TAMs are associated with enhanced tumor angiogenesis in HCC specimens [89]. It has demonstrated that TAMs activate the STAT3 signaling in hepatocellular carcinoma cell lines results in larger tumor size, intrahepatic metastasis and high recurrence rate of the tumor [90]. Additionally, it has observed that the therapeutically effects of sorafenib enhance following the tumor-associated macrophages depletion by zoledronic acid [91].

## 15. Extracellular Matrix

Hynes believed that extracellular matrix is not just “pretty fibrils” and it is more than a passive physical support [92], it is a dynamic structure and functionally regulates the cell number, morphology, movement and adhesion [93]. Such scaffold is also involved in tissue survival, growth and differentiation. Of note, the composition of ECM varies from one tissue to another. By means of versatile surface receptors, cells sense and transmit the signals from environment into the cells [94,95,96,97]. Normal composition of ECM is regulated by precise control of gene expression, the process that eventually leads to tissue homeostasis. Therefore, it would not be surprising if any changes affect the cell behaviors and develop the diseases [98,99]. To make sense, remodeling enzymes such as metalloproteinases or serine proteases are normally inactive to avoid their destructive role on ECM [100]. Unusual ECM is a hallmark of cancers [101]. Several findings have revealed that physical and biochemical composition of ECM has clearly changed in tumoral stroma such as deposition of ECM. Increased stiffness has been also observed in HCC tumors, this aspect is partly due to the overexpression of lysyl oxidase (LOX) to produce collage cross-linking with other ECM components [102]. It is necessary to mention that collagen fibrils are more oriented in cancer’s ECM than the normal one. It seems that such topography facilitates the tumor angiogenesis and invasion [102]. On the other hand, remodeler enzymes such as MMPs are also overexpressed in cancerous ECMs, and involved to physically remove the basal membrane to invade [103]. It is demonstrated that MMPs produced fragments as pro-apoptotic or pro-angiogenic effectors [104]. As previously discussed, CAFs, TAMs and endothelial cells surrounding the tissue parenchyma are the main drivers of the ECM remodeling by secretion of a variety of enzymes and cytokines. It is also important to recall that ECM components act as anchors for tissue-residing stem cells where the different signaling pathways such as FGF2 or BMP4 eventually dictate the cells to expand or differentiate. Obviously, disruption of such ecosystem potentiates the cells as cancer stem cells [102,105]. In case of HCC, in silico analysis showed that ECM-encoded genes including collagens, glycoproteins and proteoglycanes were differentially expressed between cancerous cells and corresponding normal liver [106]. It has experimentally confirmed that serum level of proteoglycanes endocan and syndecan-1 are increased in HCC patients and correlate with survival and tumor recurrence [107]. Several studies have shown that endocans actively participate in cell adhesion, proliferation and migration [108]. Altered expression of MMPs-encoding gens were observed in different human tumors showing the role of such destructive enzymes during cancer progression [103]. By means of northern blot analysis, increased expression of *MMP-9* was detected in HCC patients especially in tumor portion around the capsule. This finding demonstrated that *MMP-9* is probably involved in HCC invasion [109]. Similarly, Sun and coworker showed that high expression of *MMP-9* are strongly connected to the clinic-pathologic parameters including tumor size, capsule status, tumor stage and HCC recurrence risk [110]. It seems that hepatitis B viral HBx triggers the up-regulation of *MMP-9* through the activation the PI3K, AKT and ERK signaling pathways [111,112]. Additionally, increased expression of *MMP-9* in serum of HCC patients make it as a candidate diagnostic marker [113]. Similar pattern was observed for *MMP-2* gene [114].

## 16. Tumor-Associated Endothelial Cells (TECs)

Endothelial cells (ECs) are the fundamental cells leaning the interior face of vessel’s walls. The ECs are firmly attached to each other producing a barrier against porous vessels and bleeding. This property is partly due to presence of tight junctions between two adjacent cells. Additionally, endothelial cells are surrounded by other cells such as pericytes that make them more stable and control the vessel diameter and elasticity as well [115]. Besides, endothelial cells interact with an ECM and basement membrane proteins such as collagen, laminin and fibronectin. Such environment plays an important role during endothelial cell stability, morphogenesis, proliferation and neoangiogenesis. In the later one, basement membrane degrades and the exposure of endothelial cells to collagen triggers the new blood formation [116]. During the tumor growth, neoangiogenesis is a critical step to supply cancer cells for nutrients and oxygen. In fact, tumor progression and of course metastasis or invasion are undeniably connected to angiogenesis [117]. How the angiogenesis starts, related to the tumor environment’s changes. Such changes are produced in parallel with tumor growth. As an example, tumor environment became hypoxic and metabolic pathways of tumor cells make it acidic. These are two of important signals that induce neoangiogenesis through VEGF signaling pathway [117]. Interestingly, there are some evidences showing that endothelial cells (ECs) and their related pericytes are morphologically and genetically different with normal one. This observation was evidently against previous thoughts which knew the ECs as genetically stable cells. Studies showed tumor-associated endothelial cells (TECs) have an irregular shape, the cells are porous and leaky as they have lost their conventional tight junctions [118,119]. In an experiment on melanoma- and liposarcoma-derived cells, Hida and coworkers showed that TECs are genetically unstable. The cells are karyotyped aneuploid with a variety of structural or numerical aberrations. Hida found that TECs grew faster without any serum dependence condition [120]. There were also evidences of differential gene expression between normal and tumorous ECs in this work. On an experiment on colorectal carcinoma, Croix et al. evidenced that 64 genes are exclusively expressed in tumor-derived ECs. Some of these genes were matrix remodeler enzymes to facilitate the formation of new vessel [121]. These kinds of changes have challenged the effectiveness of angiogenesis therapy of cancers. Like other malignancies, angiogenesis play an important role during HCC progression [122]. Studies showed that VEGF—an endothelial-specific marker—has been increased in serum of patients suffering from HCC and strongly related to the degree of invasiveness, metastasis and shorter survival [123,124]. Besides, inhibition of VEGF suppresses the angiogenesis and decreases the Hepa129 and SVEC4-10 HCC cells to proliferate and growth [125].

## 17. Therapeutic Value of Tumor Microenvironment in Hepatocellular Carcinoma

As pointed above, tumor microenvironment acts as a fertile soil to grow the cancerous seeds [126]. In fact, cancer cells surrounded by different cells such as fibroblasts and inflammatory cells which are subjected to secrete a variety of growth factors, cytokines or matrix remodeling enzymes. In such media, cancer cells are potentiated to proliferate, grow or invade. This is the reason that targeted therapy of TEM is in field of attention by researchers [127]. Different strategies were taken by scientists to achieve the goal; these are either targeting the ECM components or blocking the signaling cross-talk between the cancer cells and their related stroma [128]. Targeting the components of angiogenesis and inflammatory pathways are the two well-studied processes in treatment of HCC and other malignancies and most of the developed drugs are also designed to target these paths [129]. Table 1 shows some of these drugs that are now under investigation for HCC treatment and have been nicely reviewed by Taketomi [130]. Sorafenib is one of the most efficacious drugs that are now applied for patients with advanced stage HCC. Sorafenib is multiple-kinase inhibitor capable to target VEGFR, Raf-kinase and PDGFR and suppresses cell proliferation and angiogenesis. Sorafenib has passed the phase III clinical trial to confirm its safety and tolerability [131]. Other drugs including Brivanib (targets VEGFR, FGFR), Suitinib (PDGFR, VEGFR, C-KIT, FLT-3), Lilifanib (VEGFR, PDGFR), Erlotinib (targets EGFR), Bevacizumab (targets VEGF), Cetuximab (targets EGFR), Axitinib (targets VEGFR) are in different phases of clinical trial [132]. The Phosphomannopentaose sulfate (PI-88) is another formulation to suppress HCC metastasis and recurrence through the inhibition of heparanase and sulfatase enzymes. Such inhibition can negatively influence angiogenesis in tumor cells [133]. In a randomized phase II of clinical trial, Liu and co-worker showed that dose 160mg/day of PI-88 is safe for patients who underwent surgery [134]. Another alternative is inhibition of the stromal cells that are actively connected with development of neoplastic niche. Among such cells CAFs, HSCs and TAMs are good candidates. The Sibrotuzumab is one of such drugs designing to target activated HSCs. Sibrotuzumab is promising preparation as it is nontoxic and preferentially target cancer cells [135]. Studies showed that HSCs and myofibroblasts express membrane-bounded serine proteases termed fibroblast activation protein (FAP) [136]. The FAPs are belonged to the prolyloligopeptidase gene family playing an important role in tumor biology and expresses in several solid epithelial-derived tumors such as breast [137], gastric [138] and HCC [139]. Functionally, FAPs act as ECM remodeling enzymes capable to target collagen producing biologically active fragments for the tumor growth and invasion [140]. Targeting of central signaling pathways in HCC such as TGF-β is another noteworthy approach. Transforming growth factor-β is a pleiotropic molecule mainly produced by HSCs. It is involved in different acts from ECM synthesis and remodeling to proliferation and migration. Galunisertib is an inhibitor of TGF-β which reduce the desmoplastic reaction, neoangiogenesis and intravasation in HCC [141,142].The drug is on phase II clinical trial for patients who failed to response to Sorafenib treatment [143].

## 18. Perspective and Conclusions

Despite the previous view that the tumor arose from genetically unstable cells, it has recently been confirmed that tumor microenvironment can strongly support cells that are genetically potent to show the cancer phenotype. In this way, all cellular and non-cellular fractions of tumor microenvironment prepare a neoplastic niche where the tumor can proliferate rapidly and also escape from host defense systems against damaged cells. The tumor microenvironment components also give cancer cells the opportunity to degrade, be ready for passing the basement membrane, and invade. All of these features make the tumor microenvironment a powerful target for cancer therapy. Now, scientists are trying to disturb such neoplastic niche to stop the cancer. Although a long way ahead, the results until now are promising.

## Figures and Tables

**Table 1:** TEM-based drugs in clinical trials for HCC treatment.

Drug	Molecular Targets	Phase of Clinical Trial	Referances
Sorafenib	VEGFR, PDGFR	III	[131]
Brivanib	VEGFR, FGFR	III	[144]
Sunitinib	VEGFR, PDGFR	III	[145]
Lilifanib	VEGFR	III	[146]
Axitinib	VEGFR	II	[147]
Selumetinib	MEK	II	[148]
Cetuximab	EGFR	II	[149]
Erlotinib	EGFR	III	[150,151]
Bevacizumab	VEGF	II	[152]
PI-88	HPR	II	[134]
Galuniserib	TGF-β	I	[143]
Sibrotuzumab	FAPs	I	[153]

## Abbreviations

α-SMA	α-smooth muscle actin
CAF	Carcinoma associated fibroblast
ECM	Extracellular matrix
HBV	Hepatitis B virus
HCV	Hepatitis C virus
HCC	Hepatocellular carcinoma
HSC	Human stellate cells
LPSs	Lipopolysaccharides
mTOR	Mammalian Target of rapamycin
R-ER	Rough endoplasmic reticulum
SDF-1	Stromal cell-derived factor-1
TEC	Tumor endothelial cells
TAFs	Tumor-associated fibroblasts
TAMs	Tumor-associated macrophages
TEM	Tumor microenvironment
